# Effects of Alterations in Resting-State Neural Networks on the Severity of Neuropathic Pain after Spinal Cord Injury

**DOI:** 10.3390/bioengineering10070860

**Published:** 2023-07-20

**Authors:** Eunhee Park, Jang Woo Park, Eunji Kim, Yu-Sun Min, Hui Joong Lee, Tae-Du Jung, Yongmin Chang

**Affiliations:** 1 Department of Rehabilitation Medicine, School of Medicine, Kyungpook National University, Daegu 41944, Republic of Korea; ehmdpark@knu.ac.kr (E.P.); ssuni119@naver.com (Y.-S.M.); 2 Department of Rehabilitation Medicine, Kyungpook National University Chilgok Hospital, Daegu 41404, Republic of Korea; 3 Korea Radioisotope Center for Pharmaceuticals, Korea Institute of Radiological & Medical Sciences, Seoul 01812, Republic of Korea; giantstar.jw@gmail.com (J.W.P.); geekimeun@gmail.com (E.K.); 4 Department of Radiology, School of Medicine, Kyungpook National University, Daegu 41944, Republic of Korea; leehuijoong@knu.ac.kr; 5 Department of Radiology, Kyungpook National University Hospital, Daegu 41944, Republic of Korea; 6 Department of Molecular Medicine, School of Medicine, Kyungpook National University, Daegu 41944, Republic of Korea; 7 Department of Medical & Biological Engineering, Kyungpook National University, Daegu 41944, Republic of Korea

**Keywords:** neuropathic pain, resting-state fMRI, spinal cord injury

## Abstract

Neuropathic pain (NP) following spinal cord injury (SCI) is refractory to pain control strategies, and the underlying neuronal mechanisms remain poorly understood. This study aimed to determine the brain regions engaged in maintaining a spontaneous resting state and the link between those regions and the severity of NP in patients with incomplete SCI. Seventy-three subjects (41 patients and 32 age- and sex-matched healthy controls) participated in this retrospective study. Regarding the neurological level of injury, patients with incomplete SCI experienced at-level or below-level NP. The severity of NP was evaluated using a visual analog scale (VAS), and patients were divided into mild and moderate–severe NP groups based on VAS scores. Graph theory and fractional amplitude of low-frequency fluctuation (fALFF) analyses were performed to compare resting-state functional magnetic resonance imaging (fMRI) analysis results among the three groups. Graph theory analysis was performed through a region of interest (ROI)-to-ROI analysis and then fALFF analysis was performed in the brain regions demonstrating significant differences among the three groups analyzed using the graph theory. We evaluated whether the brain regions showing significant differences using graph theory and fALFF correlated with the VAS scores. Patients with moderate–severe NP showed reduced node degree and fALFF in the left middle frontal gyrus compared with those with mild NP and healthy controls. Furthermore, patients with severe NP demonstrated increased average path lengths and reduced fALFF values in the posterior cingulate gyrus. This study found that changes in intrinsic oscillations of fMRI signals in the middle frontal gyrus and posterior cingulate gyrus were significant considering the severity of NP.

## 1. Introduction

Approximately 50% of patients with spinal cord injury (SCI) suffer from neuropathic pain (NP) [1,2]. The presence and severity of NP are associated with substantial physical and emotional functioning impairment, thereby affecting quality of life [3]. Reducing NP is essential to improve the quality of life of patients with SCI. However, provoking or relieving factors associated with NP following SCI remain poorly understood. Furthermore, NP is usually refractory to pain control strategies, such as pharmaceutical, behavioral, and neurological approaches [4,5].

Recent neuroimaging studies have indicated the possibility of anatomical and functional changes in brain regions associated with NP following SCI. In a diffusion tensor imaging study, SCI with NP showed significant differences in mean diffusivity (MD) values in pain-related areas compared to SCI without NP [6]. MD values increased in regions, such as the posterior parietal cortex, dorsolateral prefrontal cortex, anterior insula, and premotor cortex. Conversely, MD values decreased in the ventroposterior thalamus and amygdala [6]. A functional magnetic resonance imaging (fMRI) study in patients with SCI and NP demonstrated functional reorganization in the primary somatosensory cortex (S1) [7]. Furthermore, a recent study using resting-state fMRI (rs-fMRI) on an SCI animal model found that the development of mechanical hypersensitivity was strongly associated with increased functional connectivity between the thalamus and the S1 cortical regions [8]. Another rs-fMRI study investigated the mutual effects of motor- and pain-related networks in different brain regions on motor disability and NP intensity [9]. However, functional changes in brain regions associated with NP following SCI remain poorly understood. Moreover, brain regions that are related to the severity of NP following SCI are rarely investigated.

Low-frequency blood oxygenation level-dependent (BOLD) signal oscillations, which are intrinsic components of brain activity, are represented by rs-fMRI [10]. Several analyses are used to process rs-fMRI data to explore oscillatory BOLD signal dynamics associated with changes in neural activity. A graph theory analysis helps elucidate the organization of functional connections and their efficient integration of neural information in a whole brain. In a graph theory analysis, a whole-brain network is described as a graph comprising a collection of nodes and edges between nodes, revealing that the brain network is organized according to an efficient small-world organization [11,12]. A recent systematic review found that differences between patients with chronic pain and healthy controls were mainly observed in terms of the global graph-based connectivity; however, there are no particularly affected brain regions [13]. Fractional amplitude of low-frequency fluctuation (fALFF) analysis characterizes the frequency distribution of signal variance in a time series in particular brain regions [14,15]. The fALFF is an index that quantifies spontaneous neuronal activity related to the regional metabolic level of glucose [16].

In this study, we used a novel method by combining graph theory and fALFF analyses to investigate the possible alterations in brain networks associated with the severity of NP after SCI. We hypothesized that increased NP severity following SCI is related to less efficient connections in a whole-brain region assessed using graph theory analysis. Moreover, these inefficient brain regions would be associated with less spontaneous neuronal activity assessed using fALFF analysis to modulate more severe pain conditions. Therefore, this study is the first to simultaneously investigate less efficient connections and spontaneous neuronal activities at these connections. Our findings can provide a useful reference on brain responses to the severity of NP following SCI.

## 2. Materials and Methods

### 2.1. Subjects

The following subjects were included in this retrospective cross-sectional study: (1) diagnosed with SCI more than 3 months after a traumatic injury; (2) diagnosed with incomplete SCI, defined as some degree of retained motor or sensory function below the site of injury, including sacral root segments (S4–5), according to the American Spinal Injury Association Impairment Scale (AIS) [17,18]; and (3) diagnosed with persistent at- or below-level NP according to the International Association for the Study of Pain (IASP) definition [19,20]. The exclusion criteria were as follows: (1) history of nociceptive musculoskeletal pain after SCI, (2) history of traumatic brain hemorrhage or contusion on brain computed tomography, (3) history of any peripheral nervous system disorder, (4) history of any neurologic or neuropsychiatric condition, or (5) history of alcohol or drug misuse.

Seventy-three subjects (41 patients and 32 healthy controls) participated in this study. The ages of the subjects in the control (*n* = 32), mild NP (*n* = 24), and moderate–severe NP (*n* = 17) groups were 50.12 ± 13.33, 49.87 ± 14.74, and 53.82 ± 12.18 years old, respectively, with no significant age difference among the groups (*p* = 0.60). The sex of the subjects also did not differ significantly between the control (male 20, female 12), mild NP (male 15, female 9), and moderate–severe NP (male 13, female 4) groups (*p* = 0.57).

This study was approved by the Institutional Review Board of Kyungpook National University Chilgok Hospital (No. 2018-12-004).

### 2.2. Clinical Assessments

Based on the use of the International Standards for Neurological Classification of SCI worksheet, the AIS comprises an anorectal examination, a dermatome-based sensory examination, and a myotome-based motor test [17,18]. The most caudal functioning root level with intact motor and sensory functions is identified as the neurologic level of injury (NLI).

The IASP describes NP as a burning, stabbing, or shooting sensation occurring spontaneously or in response to a central nervous system injury or disease [19]. NP is characterized as either at-level or below-level NP depending on the type of NLI. At-level NP is felt in a segmental pattern anywhere along the dermatome of the NLI, whereas below-level NP is felt in more than three dermatomes beneath the dermatome of the NLI [19,20]. The average NP intensity over the previous 7 days was measured using a visual analog scale (VAS) ranging 0–100 at the time of rs-fMRI acquisition [21]. The higher the VAS score is, the more severe the NP is. The patients were then divided into two groups based on the severity of NP. A VAS score of 34 was used as the cutoff for dividing the patients into the mild and moderate–severe NP groups [22].

The Beck Depression Inventory (BDI)-II was used to measure the severity of depressive mood, and it comprised 21 items, each answer being graded from 0 to 3, with higher BDI scores indicating greater severity [23].

### 2.3. rs-fMRI Data Acquisition

The whole-brain function was acquired using a Discovery MR750w 3.0 T (GE Healthcare, Milwaukee, WI, USA) with a 24-channel head coil. Participants were given no instructions other than to close their eyes during rs-fMRI scanning to avoid falling asleep. The 240 image volumes were acquired using a T2-weighted echoplanar imaging pulse sequence with the parameters of repetition time (TR, 2000 ms), echo time (TE, 30 ms), field of view (FOV, 23 cm), matrix (64 × 64), and slice thickness:gap (4:0 mm) and no gap for resting-state imaging. T1-weighted fast spoiled gradient echo sequence (FSPGR) with a TR of 8.5 ms, TE of 3.2 ms, FA of 13°, FOV of 25.6 cm, acquisition matrix of 256 × 256, and iso-voxel resolution of 1 mm was used to obtain structural brain images.

### 2.4. rs-fMRI Data Preprocessing

Image data were preprocessed and statistically analyzed using the Statistical Parametric Mapping program (SPM12; Wellcome Centre for Human Neuroimaging, London, UK) and MATLAB (The MathWorks, Inc., Natick, MA, USA). Preprocessing included slice timing, realignment, coregistration on individual T1 structural image volume and normalization into the standard stereotaxic coordinate space (Montreal Neurological Institute). Normalized images were spatially smoothed with an 8-mm Gaussian kernel. The preprocessed rs-fMRI data were then temporally band-pass filtered (0.008–0.09 Hz) to eliminate low-frequency drift and high-frequency noise. A component-based noise correction method (CompCor) was used in the denoising part of the CONN toolbox to identify and eliminate components, such as physiological influences on fMRI data (https://www.nitrc.org/projects/conn/) (accessed on 12 March 2020) [24].

### 2.5. Graph Theory and fALFF Analyses

Graph theory and fALFF analyses were performed using the CONN toolbox. A total of 140 regions of interest (ROIs) provided by the CONN toolbox were used for the analyses. First, graph theory analysis was performed via ROI-to-ROI analysis, and the brain region exhibiting a significant difference among the three groups was confirmed using analysis of variance (ANOVA). Subsequently, fALFF analysis was performed in the brain regions demonstrating significant differences (identified using ANOVA) among the three groups analyzed using graph theory. We then investigated whether the graph theory and fALFF analyses values in the region with significant differences correlated with VAS scores (an index of pain). Finally, in regions that correlated with VAS scores, we observed whether graph theory and fALFF analyses values were correlated.

#### 2.5.1. Graph Theory Analysis

Graph theory analysis was performed within 140 ROIs provided using the CONN toolbox [25]. A network can be represented as a graph by G (*n*, *k*), indicating the number of nodes (*n*) and the number of edges (*k*). The threshold for the ROI-to-ROI connectivity matrix for each subject is at a set level (cost = 0.15, two-sided). The normalized Z-scores of raw connectivity values can be used to calculate this threshold, resulting in graphs with fixed network-level costs. Supra-threshold connectivity values are used to create an adjacency matrix that represents a graph with nodes representing ROIs and edges representing the intensity of their functional connectivity. The degree of a node is defined as the number of its connected links. The clustering coefficient of a node is defined as the ratio of the actual number of links between neighbors and the maximum possible number of links between these neighbors. The average path length is defined by the integration of a network and the easy flow of information within this network. For each node *n* in a graph G, the cost is defined as the proportion of connected neighbors, global efficiency is defined as the average inverse shortest path distance from node *n* to all other nodes in the graph G, and local efficiency is defined as the average global efficiency across all nodes in the local subgraph of node *n* [26].

The graph theory analysis of each node revealed a region with significant differences among the three groups and correlation with VAS, and this region was used for fALFF analysis. The significance level was set at *p* < 0.005 (uncorrected).

#### 2.5.2. fALFF Analysis

The rs-fMRI data with preprocessing were subjected to fALFF analysis using the CONN toolbox. The value of fALFF is equal to the sum of amplitudes within a low-frequency band divided by the sum of amplitudes across the entire frequency band [15]. To standardize raw power measures, Z-transformation was performed for the fALFF analysis, which can improve the subsequent statistical analyses on the group level. The fALFF value was obtained for the regions identified via the graph theory analysis and was used to evaluate the fALFF difference among the three groups and the correlation with VAS.

### 2.6. Statistical Analysis

All statistical analyses were performed using the SPSS software version 23 (SPSS, Inc., Armonk, NY, USA); a *p* value of <0.05 was considered statistically significant. After verifying normality by performing the Shapiro–Wilk test, one-way ANOVA was performed if normality was satisfied. For post-hoc analysis, Scheffe’s multiple comparisons were performed if equality of variance was satisfied after verification of equal variance through the Levene’s test. If the equality of variance is not satisfied, Bunnett T3 verification was performed. If the normality was not satisfied, analysis was performed using the Kruskal–Wallis *t*-test.

## 3. Results

### 3.1. General Characteristics

The VAS and BDI scores were evaluated in patients with NP. The VAS score differed significantly between the mild and moderate–severe NP groups (*p* < 0.001), whereas the BDI score did not (*p* = 0.67). The demographic and clinical characteristics of patients with incomplete SCI are summarized in Table 1 and Table S1.

### 3.2. Graph Theory Analysis According to the Severity of NP Following SCI

**Figure 1:**
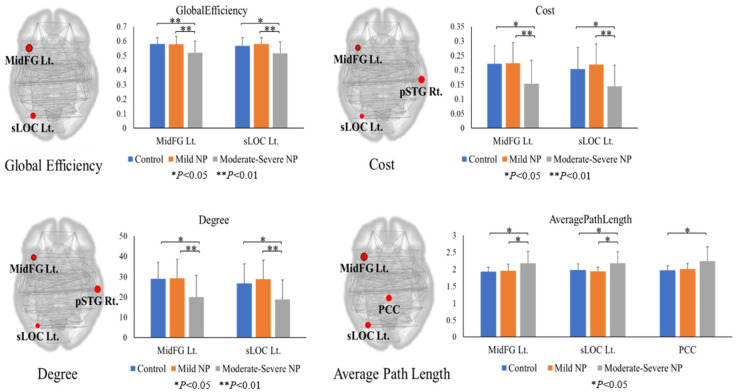
Graph theory analysis at 165 nodes. The nodes exhibited a significant difference (uncorrected *p* < 0.005) among the three groups and correlated with the VAS score (*p* < 0.05). Neuropathic pain, NP; left, Lt.; middle frontal gyrus, MidFG; superior division of lateral occipital cortex, sLOC; posterior division of cingulate gyrus, PCC; right, Rt.; posterior division of superior temporal gyrus, pSTG.

Figure 1, Table 2 and Table 3 shows the brain regions analyzed by one-way ANOVA in the three groups based on graph theory analysis (uncorrected *p* < 0.005). The left middle frontal gyrus (MidFG) and the left superior division of lateral occipital cortex (sLOC) showed differences among the three groups in global efficiency, cost, average path length, and degree. The moderate–severe NP group showed lower values than the control and mild NP groups in global efficiency, cost, and degree of the left MidFG and higher values in the average path length. No difference was observed between the control and mild NP groups. The posterior division of the cingulate gyrus (PCC) showed differences among the three groups in average path length and betweenness centrality. The moderate–severe NP group demonstrated the highest value of the average path length in the PCC area, which differed significantly from that of the control group. However, no significant difference was found between the mild NP and the other two groups.

**Figure 2:**
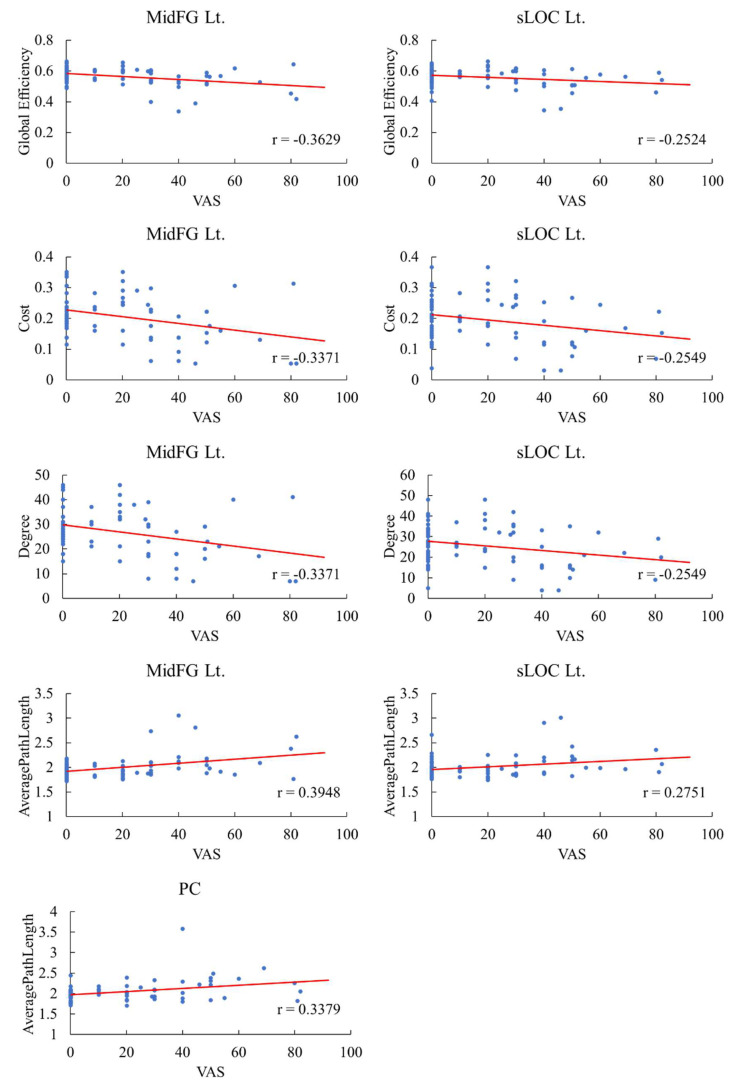
Correlation between the graph theory analysis values and VAS scores at nodes analyzed using the graph theory. Neuropathic pain, NP; visual analog scale, VAS; left, Lt.; middle frontal gyrus, MidFG; superior division of lateral occipital cortex, sLOC; posterior division of cingulate gyrus, PCC.

In the left MidFG, the global efficiency, cost, average path length, and degree showed significantly high correlations with the VAS score (*p* < 0.01), and in the left sLOC, these parameters showed negative correlations with the VAS score (Figure 2). However, the left sLOC showed a significance level of *p* < 0.05, which was lower than that of the left MidFG. In the left MidFG and left sLOC, the average path lengths demonstrated positive correlations with VAS, but other results of graph theory analysis showed negative correlations. In the PCC, the average path length was highly significantly positively correlated with the VAS score (*p* < 0.01).

### 3.3. fALFF Analysis According to the Severity of NP Following SCI

**Figure 3:**
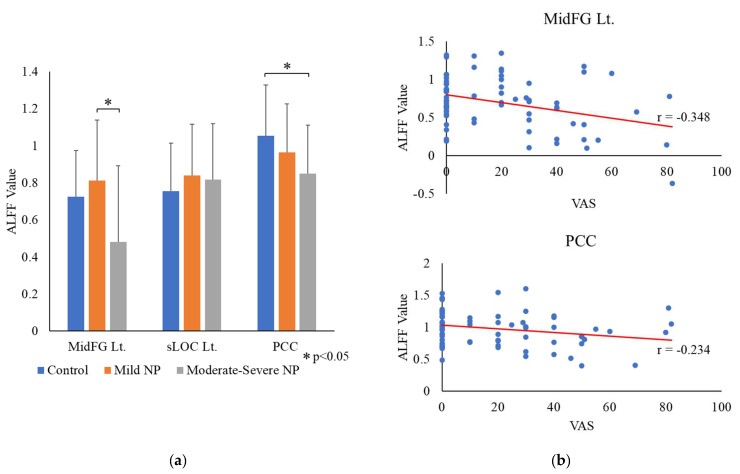
(**a**) The graph on the left shows the fractional amplitude of low-frequency fluctuation (fALFF) analysis results for three regions selected from the graph theory analysis results. (**b**) The graph on the right shows two regions with fALFF values differing among the three groups and correlating with VAS scores. Neuropathic pain, NP; visual analog scale, VAS; left, Lt.; middle frontal gyrus, MidFG; superior division of lateral occipital cortex, sLOC; posterior division of cingulate gyrus, PCC.

Figure 3 and Table 4, Table 5 and Table 6 show the fALFF analysis results for three brain regions selected from the graph theory analysis results. Among the three regions selected through graph theory analysis, the regions showing significant differences among the three groups were the left MidFG (*p* = 0.004) and PCC (*p* = 0.041). No significant difference was observed among groups in other regions. For the left MidFG region, the fALFF values were ranked in the following order of groups: mild NP < control < moderate–severe NP. The fALFF value of the moderate–severe NP group differed significantly from those of the mild NP and control groups (no significant difference was observed between the mild NP and control groups). In the PCC region, the fALFF values were ranked in the following order of groups: control < mild NP < moderate–severe NP; the fALFF values differed significantly between the control and moderate–severe NP groups but not between the control and mild NP groups or between the mild NP and moderate–severe NP groups.

The fALFF value of the left MidFG showed a high statistical level of negative correlation with the VAS score (less than *p* < 0.01, Figure 3b). Similarly, the fALFF value of the PCC showed a negative correlation with the VAS score, but the statistical level (*p* < 0.05) was lower than that of the left MidFG.

## 4. Discussion

This study found that changes in intrinsic BOLD oscillations in the MidFG and PCC were statistically significant in association with the severity of NP. Patients with moderate–severe NP showed reduced degree and fALFF in the MidFG compared with those with mild NP or healthy controls. Furthermore, patients with more severe NP had increased average path length and reduced fALFF in the PCC.

Neuroimaging studies of chronic pain have focused on nociceptive pain-processing-related brain regions. The brain regions most commonly activated by noxious stimuli are somatosensory (e.g., the S1, thalamus, and secondary somatosensory cortex), limbic (e.g., the anterior cingulate cortex and insula), and associative structures (e.g., the prefrontal cortex) [27]. Emotional and cognitive process regions, such as the dorsolateral prefrontal cortex, anterior cingulate cortex, and anterior insula [28,29], show increased activations under conditions of prolonged exposure to pain, including chronic pain induced by fibromyalgia, diabetes, and amputation. However, SCI-induced NP can be regarded as chronic pain if classified in terms of time and is not a nociceptive pain. Nevertheless, non-nociceptive pain is classified as a neurophysiological concept. A chronic lesion may cause chronic pain and continue to appear even after the injury has healed. It is known that the intensity and duration of pain is affected by adding other exacerbating factors surrounding the patient, such as stress, environment, emotions, occupation, chronic disease, and traumatic injury [30,31,32]. To the best of our knowledge, this study is the first to determine the brain regions that are highly associated with the severity of NP, affecting resting-state network efficiency and regional neural activity, after SCI.

In this study, we first analyzed the relationship between resting-state connectivity of whole-brain ROIs using graph theory and the severity of NP after SCI. Reduced global efficiency and cost values, which were interpreted in the weak potential for network integration in the left MidFG and left sLOC, were related to severe pain intensity. This finding indicates that MidFG and sLOC regions in patients with more severe NP demonstrate reduced communication with other distributed brain regions compared with healthy controls. Additionally, increased values of average path length in the left MidFG, left sLOC, and PCC were associated with increased NP severity. Higher average path length in the severe pain group was interpreted as lower efficiency in the network than that in the control group. Furthermore, reduced degree value, which was interpreted on the basis of decreased interaction with other nodes in the network in the left MidFG and sLOC, was associated with increased NP severity.

Compared with patients with mild NP or healthy controls, those with moderate–severe NP showed a reduced degree and fALFF in the MidFG. The dorsal attention network in resting-state neural networks comprises the intraparietal sulcus and the MidFG in the prefrontal cortex [33]. This network exhibits an increase in activity after presenting cues indicating where, when, or what subjects should receive attention [33,34]. Attention and cognitive function are known to modulate pain [35]. Animal studies have shown that pain stimuli interrupt attention; thus, distracting attention from that pain can reduce pain-related behaviors [36,37]. Human studies also showed that cognitive tasks induced a change in neural activity in brain regions related to pain processing, such as the thalamus, S1, anterior cingulate cortex, and insular cortex [38,39]. In addition, Wiech et al. reported that the prefrontal cortex mediated analgesic effects on expected or perceived pain [40]. In the present study, patients with moderate–severe NP showed a lower degree of connection in the MidFG than those with mild NP. A previous study proposed that a low degree of connections was linked to decreased glucose metabolism, as determined using 18F-FDG-PET [41]. Furthermore, reduced fALFF value in the MidFG might be related to reduced spontaneous neural activity in more severe pain. Prolonged NP following SCI might induce attention distraction in the MidFG to adjust to more severe pain. Further neuroimaging studies are warranted to confirm the relationship between attention-related cognitive task performance and the severity of NP in patients with SCI.

This study also showed an increased average path length and reduced fALFF in the PCC in patients with moderate–severe NP compared with those with mild NP. The average path length in graph metrics indicates brain network integration. The low efficiency of information transfer between any pair of neurons is equivalent to the increased average path length of connections between nodes [12,42]. The PCC area includes an intrinsic default mode network, and neural activity changes in the default mode network are related to chronic pain conditions, such as complex regional pain syndrome and fibromyalgia [43,44]. PCC has been closely associated with pain processing and was found to be more related to disgust than to the painful aspect of stimulation [43,44]. Particularly, the activation of ventral PCC is associated with catastrophizing and widespreadness of chronic pain [45]. Considering previous evidence, the results of our study suggest that inefficient path and reduced neuronal activity in PCC might be related to the modulation of severe NP.

Recent research demonstrates that physiotherapy may enhance the utilization of sensorimotor strategies in body representation for individuals suffering from SCI [46]. This finding implies that even when the body is affected by de-afferentation/de-efferantation, physiotherapy can modify the relationship between sensorimotor and visual aspects of body representation [46]. These alterations appear to be associated with changes in functional connectivity within and between frontal and parietal networks [47]. Furthermore, a recent study reported that alterations in intralimbic and limbostriatal connectivity were associated with the severity of neuropathic pain [48]. The study also found that the functional reorganization of cortico-cerebellar connectivity and subcortical areas varied depending on the onset time after SCI [49]. Based on these findings, the present study could contribute to the development of more comprehensive rehabilitation strategies to alleviate neuropathic pain. We propose that physiotherapy following SCI, which could affect functional neuroplastic changes, may help relieve neuropathic pain depending on the onset time of SCI. Additionally, further studies are needed to explore the relationship between spontaneous neural changes of functional reorganization after SCI, the severity of neuropathic pain, and the neuroplastic effects of post-SCI rehabilitation strategies.

This study has some limitations. First, the sample size was small; thus, this study is effectively considered a pilot. Further studies with a large population are required to conclude general outcomes in patients with NP after SCI. Second, although age- and sex-matched healthy subjects were enrolled as controls, further studies are needed to confirm the vulnerability of including patients with SCI without NP as a control group for investigating whether the current study findings are specific to individuals with NP following SCI. Finally, the interslice gap technique was not introduced in the current study. The gap technique is usually recommended for receiving good signal. However, using gap technique requires spatial interpolation when processing the imaging data, and thus, spatial uncertainty may have a chance to increase. Therefore, we decided not to use gap technique in the current study.

## 5. Conclusions

We investigated the changes in intrinsic BOLD oscillations associated with the severity of NP after incomplete SCI using graph theory and fALFF analyses. The results revealed that an increase in NP severity following SCI is related to less efficient connections, such as those represented by reduced degrees and increased average path length, and less spontaneous activity, such as those represented by reduced fALFF values, in the MidFG and PCC. Therefore, for possible therapeutic application, the findings of the current study could help determine the candidate brain regions to control severe and refractory NP using noninvasive brain stimulation.

## Figures and Tables

**Table 1:** Demographic of the incomplete spinal cord injury patients.

Group	Age	VAS	BDI
Mild NP	49.87 ± 14.74	20.58 ± 8.59	13.37 ± 8.85
Moderate–Severe NP	53.82 ± 12.18	54.35 ± 14.92	14.82 ± 11.99

Neuropathic pain, NP; visual analog scale, VAS; Beck depression inventory, BDI.

**Table 2:** The ANOVA result of graph theory analysis among three groups and the result of correlation with VAS.

Measure	ROI	ANOVA		Correlation with VAS	
		F	*p*	r	*p*
Global Efficiency	MidFG Lt.	7.35	0.001	−0.3629	0.001
	sLOC Lt.	6.45	0.002	−0.2524	0.031
Cost	MidFG Lt.	6.48	0.002	−0.3371	0.003
	sLOC Lt.	5.89	0.004	−0.2549	0.029
Degree	MidFG Lt.	6.48	0.000	−0.3371	0.003
	sLOC Lt.	5.84	0.004	−0.2549	0.029
Average Path Length	MidFG Lt.	8.01	0.000	0.3948	0.000
	sLOC Lt.	7.11	0.001	0.2751	0.018
	PCC	7.08	0.001	0.3379	0.003

Visual analog scale, VAS; left, Lt.; middle frontal gyrus, MidFG; superior division of lateral occipital cortex, sLOC; posterior division of cingulate gyrus, PCC.

**Table 3:** Post hoc analysis of graph theory results in the area where there is a statistical difference among the three groups and is correlated with VAS.

Measure	ROI	Control vs. Mild NP	Control vs. Moderate-Severe NP	Mild NP vs. Moderate-Severe NP
Global Efficiency	MidFG Lt.	0.9990	0.0030	0.0060
	sLOC Lt.	0.6970	0.0190	0.0040
Cost	MidFG Lt.	1.0000	0.0140	0.0080
	sLOC Lt.	0.7700	0.0280	0.0060
Degree	MidFG Lt.	1.0000	0.0140	0.0080
	sLOC Lt.	0.7170	0.0280	0.0060
Average Path Length	MidFG Lt.	1.0000	0.0110	0.0130
	sLOC Lt.	1.0000	0.0360	0.0140
	PCC	1.0000	0.0310	0.3130

Neuropathic pain, NP; visual analog scale, VAS; left, Lt.; middle frontal gyrus, MidFG; superior division of lateral occipital cortex, sLOC; posterior division of cingulate gyrus, PCC.

**Table 4:** In the area of graph theory analysis result, the ANOVA result of ALFF analysis among three groups and the result of correlation with VAS.

ROI	ANOVA		Correlation with VAS	
	F	p-unc	r	*p*
MidFG Lt.	5.73	0.004	−0.348	0.002
sLOC Lt.	0.71	0.491	0.102	0.387
PCC	3.33	0.041	−0.234	0.045

Visual analog scale, VAS; left, Lt.; middle frontal gyrus, MidFG; superior division of lateral occipital cortex, sLOC; posterior division of cingulate gyrus, PCC.

**Table 5:** Post hoc analysis of ALFF results in the area where there is a statistical difference among the three groups and is correlated with VAS.

ROI	Control vs. Mild NP	Control vs. Moderate-Severe NP	Mild NP vs. Moderate-Severe NP
MidFG Lt.	0.619	0.096	0.027
PCC	0.451	0.043	0.409

Neuropathic pain, NP; visual analog scale, VAS; left, Lt.; middle frontal gyrus, MidFG; posterior division of cingulate gyrus, PCC.

**Table 6:** In the area of graph theory analysis result, the ALFF value for each group.

ROI	Control	Mild NP	Moderate-Severe NP
MidFG Lt.	0.726 ± 0.247	0.813 ± 0.326	0.48 ± 0.411
sLOC Lt.	0.755 ± 0.258	0.841 ± 0.276	0.816 ± 0.303
PCC	1.055 ± 0.274	0.963 ± 0.263	0.849 ± 0.264

Neuropathic pain, NP; left, Lt.; middle frontal gyrus, MidFG; superior division of lateral occipital cortex, sLOC; posterior division of cingulate gyrus, PCC.

## Data Availability

Not applicable.

## Supplementary Materials

The following supporting information can be downloaded at: https://www.mdpi.com/article/10.3390/bioengineering10070860/s1, Table S1: Demographic and clinical characteristics of the incomplete spinal cord injury patients.
