# Supplementary material for: Effects of Alterations in Resting-State Neural Networks on the Severity of Neuropathic Pain after Spinal Cord Injury
Source: Bioengineering (Basel). 2023 Jul 20;10(7):860. doi: 10.3390/bioengineering10070860 (PMC10376439; doi:10.3390/bioengineering10070860)
Supplement: Supplementary file 1 [file bioengineering-10-00860-s001.zip › bioengineering-2468350-supplementary.pdf]

Table S1. Demographic and clinical characteristics of the incomplete spinal cord injury patients.

Patient	Sex	Age	AIS	NLI	VAS	BDI
Mild NP Group						
1	M	35	D	C2	25	10
2	M	44	D	T10	30	32
3	F	60	D	C4	29	16
4	F	70	C	L1	30	6
5	F	48	D	T9	20	18
6	M	29	D	C4	20	6
7	M	46	C	C4	10	26
8	M	26	D	C8	10	0
9	M	64	D	C2	30	28
10	M	66	D	C5	20	6
11	F	58	D	T6	20	7
12	F	70	C	T12	10	16
13	M	29	C	C5	10	9
14	M	33	C	L1	20	10
15	M	31	D	T10	30	1
16	F	65	C	T5	20	17
17	F	59	D	C4	20	10
18	M	54	D	T12	20	25
19	M	45	D	L1	20	23
20	M	37	D	C5	30	15
21	F	68	D	L2	30	3
22	M	63	D	C4	0	17
23	F	58	D	T12	10	16
24	M	39	D	C5	30	4
Mean ± SD	-	49.87±14.74	-	-	20.58±8.59	13.37±8.85
Moderate-Severe NP Group						
25	M	51	D	T10	40	6
26	M	57	D	C5	46	5
27	M	46	C	C4	55	23
28	F	51	D	C4	82	26
29	M	42	C	C4	69	9
30	F	69	D	C3	51	22
31	M	44	C	L3	50	7
32	M	64	D	C2	50	49
33	M	54	D	C5	40	7
34	M	64	D	C4	40	10
35	F	53	D	T12	80	6
36	F	69	D	C3	81	20
37	M	56	C	T11	40	15
38	M	66	D	C2	60	6
39	M	61	D	C4	50	2
40	M	20	D	C5	40	11
41	M	48	C	T12	50	28
Mean ± SD	-	53.82±12.18	-	-	54.35±14.92	14.82±11.99

Neuropathic pain, NP; american Spinal Injury Association Impairment scale, AIS; neurologic level of injury, NLI; visual analog scale, VAS; Beck depression inventory, BDI;
